# Nasopharyngeal Carriage of Methicillin-Resistant *Staphylococcus aureus* (MRSA) among Sickle Cell Disease (SCD) Children in the Pneumococcal Conjugate Vaccine Era

**DOI:** 10.3390/idr13010022

**Published:** 2021-03-01

**Authors:** Nicholas T. K. D. Dayie, Deborah N. K. Sekoh, Fleischer C. N. Kotey, Beverly Egyir, Patience B. Tetteh-Quarcoo, Kevin Kofi Adutwum-Ofosu, John Ahenkorah, Mary-Magdalene Osei, Eric S. Donkor

**Affiliations:** 1 Department of Medical Microbiology, University of Ghana Medical School, P.O. Box KB 4236, Korle Bu, Accra 00233, Ghana; dnksekoh@st.ug.edu.gh (D.N.K.S.); fcnkotey@flerholiferesearch.com (F.C.N.K.); pbtetteh-quarcoo@ug.edu.gh (P.B.T.-Q.); mmosei@ug.edu.gh (M.-M.O.); esampane-donkor@ug.edu.gh (E.S.D.); 2 FleRhoLife Research Consult, P.O. Box TS 853, Teshie, Accra 00233, Ghana; 3 Department of Bacteriology, Noguchi Memorial Institute for Medical Research, University of Ghana, P.O. Box LG 581, Legon, Accra 00233, Ghana; begyir@noguchi.ug.edu.gh; 4 Department of Anatomy, University of Ghana Medical School, P.O. Box KB 4236, Korle Bu, Accra 00233, Ghana; kadutwum-ofosu@ug.edu.gh (K.K.A.-O.); jahenkorah@ug.edu.gh (J.A.)

**Keywords:** sickle cell disease, nasopharyngeal carriage, multidrug resistant, *Staphylococcus aureus*

## Abstract

The aim of this cross-sectional study was to investigate *Staphylococcus aureus* nasopharyngeal carriage epidemiology in relation to other nasopharyngeal bacterial colonizers among sickle cell disease (SCD) children about five years into pneumococcal conjugate vaccine 13 (PCV-13) introduction in Ghana. The study involved bacteriological culture of nasopharyngeal swabs obtained from 202 SCD children recruited from the Princess Marie Louise Children’s Hospital. *S. aureus* isolates were identified using standard methods and subjected to antimicrobial susceptibility testing using the Kirby-Bauer disc diffusion method. Cefoxitin-resistant *S. aureus* isolates were screened for carriage of the *mecA*, *pvl*, and *tsst-1* genes using multiplex polymerase chain reaction. The carriage prevalence of *S. aureus* was 57.9% (*n* = 117), and that of methicillin-resistant *S. aureus* (MRSA) was 3.5% (*n* = 7). Carriage of the *mecA*, *pvl*, and *tsst-1* genes were respectively demonstrated in 20.0% (*n* = 7), 85.7% (*n* = 30), and 11.4% (*n* = 4) of the cefoxitin-resistant *S. aureus* isolates. PCV-13 vaccination (*OR* = 0.356, *p* = 0.004) and colonization with coagulase-negative staphylococci (CoNS) (*OR* = 0.044, *p* < 0.0001) each protected against *S. aureus* carriage. However, none of these and other features of the participants emerged as a determinant of MRSA carriage. The following antimicrobial resistance rates were observed in MRSA compared to methicillin-sensitive *S. aureus*: clindamycin (28.6% vs. 4.3%), erythromycin (42.9% vs. 19.1%), tetracycline (100% vs. 42.6%), teicoplanin (14.3% vs. 2.6%), penicillin (100% vs. 99.1%), amoxiclav (28.6% vs. 3.5%), linezolid (14.3% vs. 0.0%), ciprofloxacin (42.9% vs. 13.9%), and gentamicin (42.9% vs. 13.0%). The proportion of *S. aureus* isolates that were multidrug resistant was 37.7% (*n* = 46). We conclude that *S. aureus* was the predominant colonizer of the nasopharynx of the SCD children, warranting the continuous monitoring of this risk group for invasive *S. aureus* infections.

## 1. Introduction

*Staphylococcus aureus*, though a commensal, has over the years evolved into a very important human pathogen, causing mild to severe infections, including folliculitis and furunculosis, meningitis, septicaemia, pneumonia, endocarditis, and osteomyelitis [1,2]. As a commensal, it is predominantly isolated from the anterior nares, but also co-colonizes the nasopharynx with other microbiota, such as *Haemophilus influenzae*, *Moraxella catarrhalis*, and the predominant nasopharyngeal colonizer—*Streptococcus pneumoniae* [3,4,5]. Of these colonizers, *S. aureus* and *S. pneumoniae*, which antagonize each other [6,7], appear to be the most clinically significant, owing to their greater invasive disease-causing capacity [8,9,10,11] and their propensity for multidrug resistance development [12,13,14,15,16,17,18]. Colonization with these organisms is a precursor of their infections [6,19]. As *S. pneumoniae* carriage is especially high in young children [20,21,22], who bear the brunt of its high disease burden and case fatality [23,24,25], pneumococcal conjugate vaccines (PCVs) were incorporated into paediatric vaccination programmes to protect the children against *S. pneumoniae* carriage and infections [23,25,26].

However, the nasopharyngeal microbiota of vaccinated individuals and their close contacts have been reportedly altered in response to PCV introduction [27,28]. Given the antagonism between *S. aureus* and *S. pneumoniae*, there are concerns that *S. aureus* could potentially replace *S. pneumoniae* vaccine serotypes and emerge as the dominant colonizer of the nasopharynx as coverage of PCVs increases [6,7,29,30,31]. This raises legitimate concerns, as *S. aureus* is frequently implicated in respiratory tract infections, and its presence in the nasopharynx significantly predicts subsequent invasive infections [31,32]. Moreover, some studies have demonstrated that among sickle cell disease (SCD) patients, *S. aureus* holds more potential in causing invasive diseases than *S. pneumoniae* [33,34,35,36,37]. Besides, owing to the fact that antibiotics are frequently used among SCD patients as prophylaxes, due to their immunocompromised nature and consequent high predisposition to infections, selective antibiotic pressure could result, generating multidrug resistant microbes, such as methicillin-resistant *S. aureus* (MRSA) [38]. Infections with MRSA are often in tandem with extended hospital stays, increased healthcare costs, and high mortality rates [39,40,41]. Moreover, a recent study has reported that MRSA is responsible for 74.1% of all healthcare associated *S. aureus* infections and 30.1% of all community-associated infections [42]. In Ghana, there have been several MRSA outbreaks since 2012 [43]; that was the same year in which one of the PCVs—PCV-13—was introduced in the country. It is unknown whether the introduction of PCV-13 played a role in the insurgence of the MRSA outbreaks.

Most of the studies carried out on *S. aureus* and MRSA carriage appear to have focused on the occurrence of the pathogen in its ecological niche, the anterior nares [17,18,44,45,46,47]. Little attention seems to have been given to the occurrence of the pathogen in the nasopharynx, and how vaccination with PCVs influences that. The few studies that have evaluated the dynamics of the pathogen in relation to the introduction of PCVs appear to have emanated from developed countries [27,28,29,30]. Moreover, it is unknown how the introduction of PCVs has influenced the genetic diversity and antimicrobial resistance patterns of *S. aureus*. In addition, no such study seems to have been conducted among sickle cell disease patients in the country. Preliminary data to the current study, involving samples collected from healthy school children [48], reported that the nasopharyngeal carriage prevalence of *S. aureus* was high (23.2%). Evidently, the implications of an increased risk of occurrence of *S. aureus* and MRSA, with increased levels of antimicrobial resistance, in PCV-vaccinated SCD patients cannot be overemphasized, and an evaluation of the role of PCVs in the dynamics of *S. aureus* within this risk group would certainly contribute to addressing the MRSA menace in the country. This study thus aimed to investigate *S. aureus* and MRSA nasopharyngeal carriage epidemiology in relation to other nasopharyngeal bacterial colonizers among SCD children about four years into PCV-13 introduction in Ghana, including *S. aureus* antimicrobial susceptibility patterns, occurrence of the *LukF-PV* (*pvl*) and toxic shock syndrome-1 (*tsst-1*) genes among cefoxitin-resistant *S. aureus*, as well as identification of determinants of *S. aureus* and MRSA carriage.

## 2. Methods

### 2.1. Study Site, Design, and Sampling

This study was conducted at the Princess Marie Louise Children’s Hospital (PML) in Accra, the capital city of Ghana. The city is inhabited by about two million people, and has 27 hospitals (http://www.statsghana.gov.gh/ accessed on 18 June 2019). PML is the only major public hospital in Accra that primarily provides paediatric care. Its SCD clinic operates on Thursdays, and attends to an average of 20 children per day [49]. The study was cross-sectional, entailing the use of archived nasopharyngeal samples previously collected from 202 SCD children recruited between September 2016 and June 2017, in the study of Dayie et al. [50], which focused on *S. pneumoniae* carriage.

### 2.2. Laboratory Analysis of Samples and Identification of Bacteria

The nasopharyngeal swab samples were processed, and the bacterial isolates therein identified following standard bacteriological methods [51]. To begin with, the archived samples, which had been stored in skim milk-tryptone-glucose-glycerin (STGG) medium and kept at −80 °C, were brought out of the freezer and allowed to thaw at room temperature, after which they were vortexed. The specimens were pre-enriched in 5 mL of brain heart infusion broth, and after 24 h of incubation at 37 °C, inoculated with a sterile loop on 5% sheep blood, chocolate, MacConkey, and mannitol salt agars (Oxoid, Basingstoke, Hants, UK). The blood and chocolate agar plates were incubated at 37 °C in 5% CO_2_, whereas the MacConkey and mannitol salt agar plates were incubated aerobically at 37 °C. Growth promotion test was also performed after media preparation as part of the quality assurance processes by inoculating a known *S. aureus* control strain (ATCC 25923) into brain heart infusion broth and incubated at 37 °C.

The plates were examined for growth after 18–24 h of incubation, followed by sub-culturing and identification of the isolates using a composite of colonial morphology, the Gram staining, and various biochemical tests—indole, citrate, triple sugar iron, oxidase, tube coagulase, and catalase. The tube coagulase test was done by inoculating isolated colonies into each tube containing one drop of diluted plasma and nine drops of peptone water and incubated for 4–24 h. This test was quality controlled using a known *S*. *aureus* (ATCC 25923) strain as positive control and a known *Staphylococcus epidermidis* as negative control. Catalase- and coagulase-positive staphylococcal isolates were identified as *S. aureus*, and those that were catalase-positive, but coagulase-negative, were identified as coagulase-negative staphylococci (CoNS).

### 2.3. Antimicrobial Susceptibility Testing

Using the Clinical and Laboratory Standards Institute (CLSI) [52] recommendations, antimicrobial susceptibility patterns of all the *S. aureus* isolates were determined by the Kirby-Bauer disc diffusion method using the following antibiotics: gentamicin (10 µg), linezolid (30 µg), teicoplanin (30 µg), cefoxitin (30 µg), clindamycin (2 µg), tetracycline (30 µg), erythromycin (15 µg), penicillin (1 unit), and ciprofloxacin (5 µg). *S. aureus* ATCC 25923 was used as the positive control strain. An equivalent of 0.5 McFarland suspension of isolates were prepared according to the CLSI guidelines by adjusting the turbidity of the inoculum using Becton Dickinson Pheonix Spec TM Nephelometer. Within 15 min after preparation of the suspension, a sterile cotton swab was dipped into the suspension and seeded on sterile, dried Muller Hinton plates in order to obtain semi-confluent growth. After 15 min, using sterile forceps, the various antimicrobial discs listed above were aseptically positioned on the seeded plates. The inoculated Muller Hinton agar plates were incubated aerobically for 18–24 h at 37 °C within 15 min after the application of the antimicrobial discs. Isolates that displayed resistance to cefoxitin, showing zones of inhibition less than 22 mm, were phenotypically identified as MRSA, whereas cefoxitin zones of inhibition greater than or equal to 22 mm were phenotypically identified as methicillin-sensitive *S. aureus* (MSSA). The presumptively identified MRSA isolates were subsequently confirmed as MRSA by polymerase chain reaction (PCR) targeting the *mecA* gene and screened for carriage of the *pvl* and *tsst-1* genes.

### 2.4. Molecular Analyses

Crude DNA was extracted from overnight cultures of the isolates. For each isolate, a loopful of pure culture was harvested and emulsified in 200 µL nuclease-free water in a sterile 2 mL Eppendorf tube. The emulsion was vortexed and heated in a heat block compartment at 100 °C for 10 min, frozen at −20 °C for 10 min, and centrifuged at 3000× *g* for 10 min. The supernatant containing the bacterial DNA was aliquoted into a new, labelled Eppendorf tube, stored at −20 °C, and used as a DNA template for multiplex PCR [53].

The multiplex PCR involved the use of primer sets that targeted the *mecA*, *pvl*, and *tsst-1* genes, as described by Larsen et al. [54]. The primers were reconstituted by adding specific volumes of nuclease-free water, and vortexed vigorously for even mixing, according to the manufacturer’s instructions, to make 100 mM of stock solution, which was then stored at −20 °C. A working solution of 200 µL volume of each primer was made by adding 180µL of nuclease-free water to 20 µL of stock primer solution. The mixture was vortexed, centrifuged, and stored at −20 ℃. The primer mix containing the forward and reverse primers of each of the genes (*mecA, pvl*, and *tsst-1*) was prepared according to the dilutions in Table 1.

Amplification was performed in a Veriti 96-well thermal cycler. The amplicons (PCR products) were visualized using 2% agarose gel electrophoresis, and the band sizes were compared to that of a DNA marker (100 bp ladder from New England BioLabs, Ipswich, MA, USA). In-house *mecA*-, *pvl*-, and *tsst-1*-harbouring *S*. *aureus* isolates were used as positive controls for the *mecA*, *pvl*, and *tsst-1* genes, respectively, and nuclease-free water was used as negative control. Table 2 presents a list of primers and their sequences used for the polymerase chain reaction.

### 2.5. Data Analysis

The data were analyzed with the aid of Statistical Products and Services Solutions (SPSS), version 25. The sociodemographic and clinical data of the study participants were summarized using descriptive statistics. Independent samples Chi-square and point biserial correlation tests, followed by binary logistic regression, were used to determine predictors of *S. aureus* and MRSA colonization among the study participants. The significance of each of the predictors was determined by computing its *p*-value, odds ratio, and 95% confidence interval; predictors were deemed statistically significant if their *p*-values fell below 0.05.

### 2.6. Ethical Approval

The study was given approval by the Ethical and Protocol Review Committee of the College of Health Sciences, University of Ghana, with protocol identification number “CHS-Et/M.9-P 4.3/2015-2016”.

## 3. Results

### 3.1. Sociodemographic and Clinical Features of the Study Participants

The age of the participants ranged from 1 to 13 years (mean and standard deviation: 3.4 ± 1.9). Additionally, more than half of them were above five years of age (53.5%), were males (55.0%), and lived in compound houses (63.9%). Almost all (93.6%) of them were enrolled in school. The sociodemographic characteristics of the study participants are displayed in Table 3.

The clinical characteristics of the study participants displayed in Table 4 demonstrate that almost half (44.6%) of the children were vaccinated with PCV-13, and less than one-third had each had runny nose (30.7%), blocked nose (23.8%), productive cough (20.3%), ear infection (1.5%), and other conditions.

### 3.2. Bacterial Flora Colonizing the Nasopharynx of the SCD Children

Bacterial pathogens isolated from the nasopharynx of the participants are presented in Table 5. The carriage prevalence of MRSA and MSSA were 3.5% and 55.0%, respectively, comprising a composite *S. aureus* carriage of 57.9% (dual *S. aureus* carriage was observed among five participants, with one of these having dual MRSA-MSSA carriage and four having dual MSSA-MSSA carriage). *S. aureus* was thus the commonest colonizer, followed by *Streptococcus pneumoniae* (39.1%), coagulase negative *Staphylococci* (CoNS) (22.1%), and alpha haemolytic streptococci (19.8%). The rest of the isolates (Coliforms, Diphtheroids, *Enterococcus* spp., *Klebsiella oxytoca*, and *Pseudomonas* spp.) rarely colonized the study participants (1.5% and below). Moreover, the prevalence of *S. aureus* was significantly higher than that of *S. pneumoniae* (*p* < 0.0001).

### 3.3. Occurrence of Virulence Genes among the Cefoxitin-Resistant S. aureus Isolates

**Figure 1:**
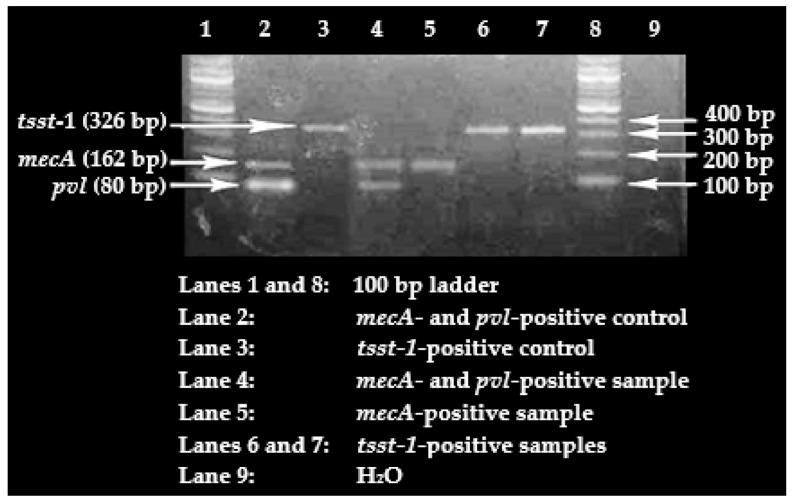
Agarose gel electrophoresis pattern for multiplex PCR amplification product of *mecA, pvl*, and *tsst-1* genes.

As observed in Table 6, of the 35 cefoxitin-resistant *S. aureus* isolates obtained in the current study, *mecA* gene carriage was confirmed in 20.0% (*n* = 7); the *pvl* and *tsst-1* genes were demonstrated in 85.7% (*n* = 30) and 11.4% (*n* = 4), respectively. Figure 1 is a representation of the agarose gel electrophoresis of the multiplex PCR products of the three genes whose presence were screened for.

### 3.4. Determinants of S. aureus Nasopharyngeal Carriage

As observed in Table 7, colonization with CoNS (*OR* = 0.044, *p* < 0.0001) and PCV-13 vaccination (*OR* = 0.356, *p* = 0.004) each appeared to protect the study participants against *S. aureus* colonization. However, none of these and other features of the participants emerged as a determinant of MRSA colonization.

### 3.5. Antimicrobial Resistance Patterns of the MSSA and MRSA Isolates

**Figure 2:**
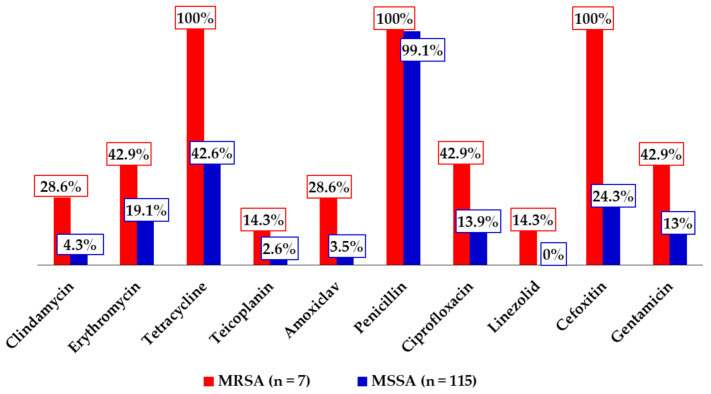
Antimicrobial resistance patterns of the methicillin-resistant *S. aureus* (MRSA) and methicillin-sensitive *S. aureus* (MSSA) isolates.

Penicillin was the antimicrobial to which the *S. aureus* isolates in the current study displayed the highest resistance (100% for MRSA, and 99.1% for MSSA). On the other hand, linezolid recorded the lowest composite resistance (14.3% for MRSA, and 0.0% for MSSA). Moreover, the MRSA isolates recorded higher tetracycline (100% vs. 42.6%), erythromycin (42.9% vs. 19.1%), amoxiclav (28.6% vs. 3.5%), ciprofloxacin (42.9% vs. 13.9%), and gentamicin (42.9% vs. 13.0%) resistance in comparison to the MSSA isolates. The proportion of *S. aureus* isolates that were multidrug resistant was 37.7% (*n* = 46). Details of the antimicrobial resistance rates can be found in Figure 2.

## 4. Discussion

This study investigated *S. aureus* nasopharyngeal carriage epidemiology in relation to other nasopharyngeal bacterial colonizers among SCD children about five years into PCV-13 introduction in Ghana. It is the first to conduct such an evaluation among SCD children post-PCV-13 introduction in the country, thus providing insights into dynamics of PCV- and SCD-induced *S. aureus* nasopharyngeal carriage epidemiology.

The isolated bacterial flora colonizing the nasopharynx of the SCD children belonged to both the Gram-positive and Gram-negative categories, comprising *S. aureus*, CoNS, *Streptococcus pneumoniae*, alpha haemolytic streptococci, Diphtheroids, *Enterococcus* spp., *Pseudomonas* spp., and *Klebsiella oxytoca*. While the other bacteria isolated were present at low numbers, MRSA and MSSA prevalence were 3.5% and 55.0%, respectively, translating to *S. aureus* domination among the isolated nasopharyngeal colonizers at a prevalence of 57.9%. It is noted that Dayie et al. [50] (the parent study) reported a significantly lower carriage prevalence of *S. pneumoniae* (39.1%) compared to the carriage prevalence of *S. aureus* observed in the current study (57.9%). Interestingly, the nasopharynx is the ecological niche of *S. pneumoniae*, but not *S. aureus*. Hence, the significantly higher carriage prevalence of *S. aureus* relative to *S. pneumoniae* among the participants, as well as the presence of the other nasopharyngeal colonizers in low numbers, suggest that the modification of the nasopharyngeal microbiota resulting from PCV-13 vaccination led to nasopharyngeal proliferation of *S. aureus* as the dominant colonizer. The observations concurrently demonstrate that PCV-13 vaccination was effective in decolonizing the study participants of vaccine-captured serotypes of *S. pneumoniae*. 

The *S. aureus* carriage prevalence recorded in the current study is higher than the post-PCV-13 vaccination anterior nasal carriage prevalence reported by Appiah et al. [18] among a cohort of SCD children (33%) and the 44.9% nasal carriage prevalence reported by Donkor et al. [19] among HIV-infected children, another immunologically challenged population, who were also studied during the post-PCV-13 vaccination era. The nasopharyngeal carriage prevalence observed in this study is higher than those reported in other countries, such as Ethiopia, The Gambia, Iran, and Portugal (10.3–30.6%) [5,56,57,58].

That *S. aureus* carriage was higher in this study than it was in the anterior nares of the SCD children in Appiah et al.’s [18] study and HIV-infected children in Donkor et al. [17] study seems to confirm an insurgence of *S. aureus* in the nasopharynx, as asserted above. This is because carriage is expected to be higher in the anterior nares (the anatomical site sampled by Appiah et al. [18] and Donkor et al.’s [17]), as it is the ecological niche of *S. aureus*, rather than the nasopharynx. It is possible that slight variations between the study population of the current study and each of the other two populations in question may account for this disparity. Indeed, should information on concurrent nasal carriage prevalence be available alongside the reported nasopharyngeal carriage prevalence, a more conclusive deduction could have been made. That notwithstanding, the higher *S. aureus* nasopharyngeal carriage prevalence of this study than the nasal carriage prevalence reported by Donkor et al. [17] also supports the deduction that *S. aureus* is becoming established in the nasopharynx. This claim is strengthened by the known proneness of HIV-infected persons to *S. aureus* nasal carriage [17,59].

Appiah et al.’s [18] study, although also conducted in the PCV-13 era, focused on nasal carriage of staphylococci, and hence it is not possible to glean from it the diversity of the colonizing bacteria for the purpose of comparing with the diversity observed in the current study. Another carriage study conducted in the study area [60], entailing surveillance of an additional organism to *S. aureus* among SCD patients, but done prior to the PCV-13 era, focused only on *S. pneumoniae* carriage, and its study design was atypical—it was an amalgam of two sub-studies, one involving a haemoglobin SS-haemoglobin AA case-control nasal carriage study focusing on *S. aureus* only, and the other involving a different cohort of SCD children who were studied for nasopharyngeal carriage of *S. pneumoniae* exclusively. Therefore, that study also did not provide data with which appropriate comparisons could be made in the context of colonizing nasopharyngeal bacterial flora.

Only 20% of the cefoxitin-resistant *S. aureus* isolates were confirmed to carry the *mecA* gene, meaning that 80% of these isolates lacked the presence of the gene. This is of concern, given that in resource-poor settings, such as Ghana, clinical laboratories generally base the identification of MRSA on oxacillin or cefoxitin resistance. Even though it is possible that the cefoxitin-resistant, *mecA*-negative, *S. aureus* isolates may be harbouring other variants of the *mec* gene, such as *mecC*, a significant proportion (80%) lacked the gene. This could be an indictment on MRSA diagnosis that is exclusively based on phenotypic detection. Concerns similar to these have been raised elsewhere [61]. It may however be argued that this apparent flaw in MRSA diagnosis in resource-poor settings probably has an invariably little impact in clinical settings, particularly, when such laboratory investigations are focused on guiding the selection of an antimicrobial agent with a contemporarily demonstrated effectiveness against an infecting pathogen for the purpose of achieving success in treatment. Hence, in that case, phenotypic demonstration of methicillin resistance would suffice in ruling out the use of certain antimicrobial regimens. The flaw, nonetheless, becomes relevant when such clinical data are submitted directly to epidemiological studies involving MRSA surveillance, or published to reflect MRSA burden in hospitals, in which cases the data may be deemed misleading. Accordingly, it is important to prioritize the upgrade of clinical laboratories in resource-poor settings so as to ensure the use of present-day techniques, such as molecular detection methods, in diagnosis.

The high proportion of *pvl* carriage was not surprising, as immunosuppression is known to predispose individuals to a higher risk of *pvl*-positive *S. aureus* carriage [62]. Besides this fact, Ghana, like other countries in Africa, is a *pvl*-positive *S. aureus*-endemic region, with the high occurrence observed even among MSSA isolates. For instance, Eibach et al. [63] reported the *pvl* gene to be present in 58% of the MSSA isolates recovered in their study conducted in Kumasi, Ghana. Devine et al. [64] also reported a *pvl* carriage proportion of 72.1% in Nigeria. That said, the high proportion of *pvl*-positive nasopharyngeal *S. aureus* colonizers is of immense clinical significance, as it indicates that this cohort of SCD patients, a known immunocompromised population, would have a higher risk of developing invasive infections with *S. aureus* strains that could cause polymorphonuclear leucocyte lysis and tissue necrosis owing to their borne virulence determinant—*pvl* [65,66,67,68].

Based on available literature published in peer-reviewed journals, this study is the first to evaluate *tsst-1* carriage among *S. aureus* strains recovered from SCD patients in Ghana. A similar proportion—9.8%—was reported in Nigeria [64]. The low carriage of the gene may mean that only a few of these SCD patients may be prone to developing the toxic shock syndrome.

It is quite interesting that having previously received PCV-13 vaccination appeared to protect the study participants against *S. aureus* colonization. This is because PCV-13, like other PCVs, was designed to specifically curtail *S. pneumoniae* carriage in the nasopharynx [69,70], and hence logically, it is feared that it would cause an insurgence of *S. aureus* nasopharyngeal carriage, given the *S. aureus*–*S. pneumoniae* antagonism [6,7,71]. Thus, the apparent unusual protective effects observed with regard to PCV-13 vaccination in this cohort is most likely not directly linked to the PCV-13 itself, but must have been mediated by secondary factors, such as the presence of CoNS, which was reported at a high rate in the current study. CoNS are also known to be antagonistic to *S. aureus*, by virtue of the CoNS-produced autoinducing peptide [72]. In fact, in the current study, CoNS colonization was observed to protect against *S. aureus* colonization. Besides the potential contribution of CoNS, other members of the nasopharyngeal microbiome, which may have been missed by the culture methods relied on in the current study, may have a bearing on this seeming protectiveness of PCV-13 against *S. aureus* carriage. Future studies could employ high-throughput sequencing, such as 16S ribosomal ribonucleic acid (rRNA) sequencing, to further investigate the dynamics introduced by CoNS colonization as well as other members of the nasopharyngeal microbiome to this indirect and atypical PCV-13-*S. aureus* relationship.

The highest antimicrobial resistance rate was recorded against penicillin, which was expected, as the antibiotic is routinely used among SCD individuals for prophylactic purposes against *S. pneumoniae* infections, has been in wide circulation for years, and is easily accessible in the country. Moreover, other *S. aureus* studies have reported similar resistance rates against it [17,18,47,73]. The low resistance rates recorded against linezolid was also expected, as the antibiotic does not have a wide coverage in the country, and previous studies conducted on *S. aureus* have reported comparable rates [17,74]. The observed rate is however at odds with the 30% resistance rate recorded among anterior nasal *S. aureus* isolates obtained in the above-cited recent study of Appiah et al. [18] among SCD children. That notwithstanding, the low rate signals that linezolid can still be administered in the treatment of MRSA infections. The high rate of tetracycline resistance, although alarming, lacks clinical significance, as the drug is rarely used in clinical practice. Moreover, the high resistance rates demonstrated by the MRSA isolates against erythromycin, amoxiclav, ciprofloxacin, and gentamicin relative to the MSSA isolates is consistent with a key attribute of MRSA—resistance to several antibiotic groups in routine use [12,75,76]. The 37.7% proportion of multidrug resistance observed in this study appears to be higher than those reported with regard to *S. aureus* in some studies in the country (3.2–16.7%) [16,48,73], whereas strikingly lower than others (62.3–100%) [17,18], and although it may reflect some beneficial effects of campaigns against irrational antimicrobial use, it synchronously underscores the need to intensify these campaigns, particularly among risk groups for *S. aureus* carriage and infections, such as SCD patients.

This study was limited by a few factors. First, the absence of information on concurrent *S. aureus* nasal carriage prevalence alongside the reported nasopharyngeal carriage prevalence makes the assertion of *S. aureus* insurgence of the nasopharynx a bit less conclusive. Furthermore, screening for virulent genes was restricted to the cefoxitin-resistant *S. aureus* isolates due to financial constraints, and hence, the study could not provide information on the proportion of *S. aureus* isolates that harboured the screened virulent genes.

## 5. Conclusions

We conclude that of the bacterial flora isolated from the nasopharynx of the sickle cell disease children, *S. aureus* was the predominant colonizer. Nasopharyngeal carriage prevalence of *S. aureus* and MRSA were 57.9% and 3.5%, respectively, suggesting an insurgence of *S. aureus* in the nasopharynx; the distribution of virulence genes among the cefoxitin-resistant *S. aureus* isolates was: 20% *mecA*, 85.7% *pvl*, and 11.4% *tsst-1*. Furthermore, no determinant of MRSA colonization was identified; colonization with coagulase-negative staphylococci and PCV-13 vaccination were each found to protect the SCD children against *S. aureus* colonization. The highest and lowest antimicrobial resistance rate were recorded against penicillin and linezolid, respectively, with 37.7% of the *S. aureus* isolates displaying multidrug resistance.

It is recommended that further studies be conducted on the *S. aureus* isolates obtained in this study to determine their sequence and *spa* types via whole-genome sequencing. In addition, this study needs to be extended to other risk groups, with concurrent sampling of the anterior nares and nasopharynx. Moreover, it is important to carry out continuous surveillance of the pathogen nationwide and intensify public health education against indiscriminate antibiotic use. There is also a need to monitor SCD patients for invasive bacterial diseases due to *S. aureus*.

## Figures and Tables

**Table 1:** Primer mix preparations for *mecA*, *pvl*, and *tsst-1* genes.

Primer	Forward (µL)	Reverse (µL)	Concentration (µM)
*mecA*	45	45	0.45
*pvl*	100	100	1
*tsst-1*	100	100	1

Volume of nuclease-free water = 510 µL; Total reaction volume = 1000 µL.

**Table 2:** Primers used for multiplex PCR screening of *mecA*, *pvl*, and *tsst-1* genes.

Gene	Primer	Sequence	Size	Reference
*mecA*	P4	TCCAGATTACAACTTCACCAGG	162 bp	Larsen et al. [54]
	P7	CCACTTCATATCTTGTAACG		
*pvl*	Forward	GCTGGACAAAACTTCTTGGAATAT	80 bp	Larsen et al. [54]
	Reverse	GATAGGACACCAATAAATTCTGGATTG		
*tsst-1*	Forward	ACCCCTGTTCCCTTATCATC	326 bp	Mehrota et al. [55]
	Reverse	TTTTCAGTATTTGTAACGCC		

**Table 3:** Sociodemographic characteristics of the study participants (*n* = 202).

Characteristics	Frequency	Percentage (%)
*Age groups*		
<5 years	94	46.5
5–9 years	75	37.1
10–15 years	33	16.4
*Gender*		
Male	111	55.0
Female	91	45.0
*Type of residence*		
Compound house	129	63.9
Self-contained	73	36.1
*Number of persons per household*		
<5	71	35.1
5–10	118	58.4
11–20	12	5.9
≥21	1	0.5
*Others*		
School attendance	189	93.6
Exposure to passive smoking	0	0.0

Mean age = 3.4 ± 1.90 years; Mean number of persons per household = 6 ± 3.

**Table 4:** Clinical characteristics of study participants (*n* = 202).

Characteristics	Frequency	Percentage (%)
PCV-13 vaccination	90	44.6
Asthma	7	3.5
Pneumonia	14	6.9
Ear infection	3	1.5
*Respiratory symptoms*		
Sore throat	18	8.9
Chest pains	16	7.9
Runny nose	62	30.7
Blocked nose	48	23.8
Productive cough	41	20.3
Difficulty in breathing	10	5.0

PCV-13 refers to pneumococcal conjugate vaccine 13-valent.

**Table 5:** Prevalence of isolated nasopharyngeal colonizers.

Bacterial pathogen	Number **	Prevalence (%)
*S. aureus* ^##^	117	57.9
MRSA	7	3.5
MSSA	111	55.0
CoNS	48	23.8
*Streptococcus pneumoniae* ^++^	79	39.1
Alpha haemolytic streptococci	40	19.8
Coliforms	3	1.5
Diphtheroids	2	1.0
*Enterococcus* spp.	2	1.0
*Klebsiella oxytoca*	1	0.5
*Pseudomonas* spp.	1	0.5

** Number refers to number of colonized participants; ^##^ Five participants had dual *S. aureus* carriage (Dual MRSA-MSSA carriage [*n* = 1], Dual MSSA-MSSA carriage [*n* = 4]); ^++^ Extracted from Dayie et al. [50].

**Table 6:** Virulence genes among the cefoxitin-resistant *S. aureus* isolates.

Gene	Carriage (N)	Percentage (%)
*mecA*	7/35	20.0
*pvl*	30/35	85.7
*tsst-1*	4/35	11.4

**Table 7:** Determinants of *S. aureus* colonization.

Determinant	Odds Ratio	95% *CI*	*p* Value
Colonization with CoNS	0.044 *	0.017–0.117	<0.0001
PCV-13 vaccination	0.356 *	0.176–0.718	0.004

* Significant at 0.05 alpha level; N/A = Not applicable.

## Data Availability

The data presented in this study are available upon reasonable request from the corresponding author via ntkddayie@ug.edu.gh.
